# Human variant of scavenger receptor BI (R174C) exhibits impaired cholesterol transport functions

**DOI:** 10.1016/j.jlr.2021.100045

**Published:** 2021-02-09

**Authors:** Sarah C. May, Jacqueline S. Dron, Robert A. Hegele, Daisy Sahoo

**Affiliations:** 1 Department of Biochemistry, Medical College of Wisconsin, Milwaukee, WI, USA; 2 Robarts Research Institute, Western University, London, Ontario, Canada; 3 Department of Biochemistry, Schulich School of Medicine and Dentistry, Western University, London, Ontario, Canada; 4 Department of Medicine, Schulich School of Medicine and Dentistry, Western University, London, Ontario, Canada; 5 Department of Medicine, Medical College of Wisconsin, Milwaukee, WI, USA; 6 Cardiovascular Center, Medical College of Wisconsin, Milwaukee, WI, USA

**Keywords:** receptors/lipoprotein, cholesterol efflux, selective uptake, dyslipidemias, human genetics, Arg-174, arginine-174, Asp-185, aspartate-185, CADD, Combined Annotation Dependent Depletion, CHE, [^3^H]cholesteryl hexadecyl ether, ExAC, Exome Aggregation Consortium, MAF, minor allele frequency, MFI, mean fluorescence intensity, PFOA, perfluorooctanoic acid, SR-BI, scavenger receptor class B type I

## Abstract

HDL and its primary receptor, scavenger receptor class B type I (SR-BI), work together to promote the clearance of excess plasma cholesterol, thereby protecting against atherosclerosis. Human variants of SR-BI have been identified in patients with high HDL-cholesterol levels, and at least one variant has been linked to cardiovascular disease. Therefore, while often regarded as beneficial, very high levels of HDL-cholesterol may result from impaired cholesterol clearance through SR-BI and contribute to cardiovascular risk. In this study, we characterized the function of a rare human variant of SR-BI, resulting in the substitution of arginine-174 with cysteine (R174C), which was previously identified in a heterozygous individual with high levels of HDL-cholesterol. We hypothesized that the R174C-SR-BI variant has impaired cholesterol transport functions, which were assessed in COS-7 cells after transient transfection with full-length WT or R174C-SR-BI. Although R174C-SR-BI was expressed at levels comparable to the WT receptor, HDL binding, cholesteryl hexadecyl ether uptake, free cholesterol efflux, and modulation of membrane cholesterol were disrupted in the presence of R174C-SR-BI. We further examined the role of salt bridges as a potential mechanism for R174C-SR-BI dysfunction. If translatable, this human variant could lead to increased plasma HDL-cholesterol levels, impaired cholesterol clearance, and increased cardiovascular disease risk.

Heart disease remains the leading cause of mortality in the United States (1). The most common type of heart disease, coronary heart disease (2), results from atherosclerosis or the accumulation of excess cholesterol in plaques within the arteries, which narrows the vessels and impairs blood flow. Long-standing epidemiological evidence from the Framingham Heart Study suggests that HDL-cholesterol levels are inversely related to coronary heart disease risk (3). HDL is thought to protect against atherosclerosis, in part, by effluxing free cholesterol out of peripheral cells (e.g., macrophages in the artery wall) and transporting cholesterol (upon esterification) to the liver for excretion from the body (reviewed in (4)). This process is known as reverse cholesterol transport and is facilitated by the HDL receptor, scavenger receptor class B type I (SR-BI).

SR-BI is abundantly expressed in liver and steroidogenic tissues (5, 6) and, to a lesser extent, in macrophages (7, 8, 9) and other cell types (reviewed in (10)). SR-BI localizes to the cell surface, where its large extracellular domain binds with high affinity to HDL (5). Based on Kyte-Doolittle hydropathy analyses (11, 12), SR-BI is predicted to have two membrane-spanning domains with two short N- and C-terminal tails that reside intracellularly. Importantly, SR-BI facilitates the bidirectional movement of cholesterol between cells and HDL particles (5, 13), thus acting at both key stages of reverse cholesterol transport to promote atheroprotection.

Knockout of SR-BI in an atherosclerotic apoE-deficient mouse model leads to accelerated development of atherosclerosis (14), confirming an atheroprotective role for SR-BI. Unexpectedly, SR-BI^−/−^ mice, which are more susceptible to atherosclerosis, have higher levels of HDL-cholesterol (15). The most likely explanation for this paradoxical finding is that SR-BI^−/−^ mice are unable to properly dispose of plasma cholesterol, resulting in the accumulation of HDL-cholesterol that cannot be effectively cleared from circulation. Thus, high HDL-cholesterol levels could be a biomarker of impaired cholesterol clearance.

Human variants of SR-BI have been associated with increased HDL-cholesterol levels. One such variant, P297S, showed impaired HDL-cholesteryl ester uptake levels (16). Brunham *et al.* (17) identified two additional human SR-BI variants, S112F and T175A, which also exhibited impaired cholesterol transport functions, as measured in vitro (18). The dysfunctional human variant P376L was further linked to increased cardiovascular disease risk (19). An Icelandic group identified three human SR-BI missense variants (G319V, V111M, and V32M); however, there was no correlation with cardiovascular disease risk (20).

In a previously published study (21), 119 individuals ascertained from a lipids clinic with elevated HDL-cholesterol levels were sequenced in an attempt to identify causative variants. Here, we describe a single patient from this cohort who had elevated HDL-cholesterol levels and was found to carry a heterozygous variant of *SCARB1* (gene encoding SR-BI) that results in the substitution of arginine-174 (Arg-174, R) with cysteine (Cys, C) (R174C). Based on the high HDL-cholesterol levels of the patient, we hypothesized that the R174C-SR-BI variant has impaired cholesterol transport functions. Our results indicate that the R174C mutation leads to reduced cholesterol transport, suggesting that its ability to clear cholesterol from circulation may be compromised.

## Materials and Methods

### Materials

COS-7 cells were obtained from ATCC (Manassas, VA). Rabbit polyclonal antibodies targeting the C-terminal region of SR-BI (amino acids 450–509; NB400-101) or near-C-terminal extracellular domain (amino acids 230–380; NB400-134) were purchased from Novus Biologicals (Littleton, CO). The anti-rabbit-GAPDH (#2118) antibody was obtained from Cell Signaling Technology (Danvers, MA). HRP-conjugated donkey-anti-rabbit-IgG secondary antibody was purchased from GE Healthcare Life Sciences (Marlborough, MA). [^125^I]sodium iodide, [^3^H]cholesteryl hexadecyl ether (CHE), and [^3^H]cholesterol were purchased from PerkinElmer (Waltham, MA). Human HDL and ACAT inhibitor (Sandoz 58-035) were obtained from MilliporeSigma (Burlington, MA). Recombinant cholesteryl ester transfer protein was from Roar Biomedical (New York, NY). Cholesterol oxidase from *Streptomyces sp.* and TLC standards (cholesterol, 4-cholesten-3-one, and cholesteryl oleate) were obtained from Sigma-Aldrich (St. Louis, MO). FuGENE 6 transfection reagent was obtained from Promega (Madison, WI). EZ-Link Sulfo-NHS-LC-Biotin and DiI-LDL were purchased from Thermo Scientific (Waltham, MA). DiI-HDL was obtained from Kalen Biomedical (Germantown, MD). All other reagents were of analytical grade.

### Genetic analyses

The patient's genomic DNA was isolated from blood using the Puregene® DNA Blood Kit (Gentra Systems, Qiagen Inc., Mississauga, Ontario, Canada). In adherence to the Declaration of Helsinki, the patient provided written informed consent for the collection of personal data and DNA with approval from the Western University (London, Ontario, Canada) ethics review board (no. 07290E). The sample was then indexed and enriched using the Nextera® Rapid Capture Custom Enrichment Kit “LipidSeq” design (22). Sequencing was done using an Illumina MiSeq personal sequencer (Illumina, San Diego, CA) at the London Regional Genomics Centre (http://www.lrgc.ca/). The generated FASTQ files were imported into CLC Bio Genomics Workbench (version 7.5; CLC Bio, Aarhus, Denmark) for read alignment to the human reference genome (hg19) and variant calling. Identified variants were annotated using ANNOVAR (23) and flagged for follow up if they met the following criteria: had a minor allele frequency (MAF) of <1% or missing in the Exome Aggregation Consortium (ExAC) database (24) (now merged into the gnomAD database; https://gnomad.broadinstitute.org/); and had an in silico prediction of deleterious or damaging according to at least half of the available predictions from Polymorphism Phenotyping (version 2; PolyPhen2; http://genetics.bwh.harvard.edu/pph2/), Sorting Intolerant From Tolerant (http://sift.jcvi.org/), MutationTaster (http://www.mutationtaster.org/), or Combined Annotation Dependent Depletion (CADD; http://cadd.gs.washington.edu/score).

### SR-BI expression vectors

Single- or double-point mutations for R174C, R174K, R174D, D185R, or R174D/D185R were designed in the coding region of the human *SCARB1* gene (herein referred to as SR-BI), which was previously cloned into the pcDNA3 expression vector (Invitrogen) (18). Top Gene Technologies (Pointe-Claire, Quebec, Canada) performed cloning, site-directed mutagenesis, and sequencing to verify mutations.

### Cell culture and transfection

COS-7 cells were maintained in DMEM supplemented with sodium pyruvate, penicillin, streptomycin, and FBS at 37°C/5% CO_2_. Upon reaching ∼70% confluency, cells in 10 cm dishes were transiently transfected with 10 μg empty plasmid vector (pcDNA3) or plasmids encoding WT or mutant SR-BI receptors, using the FuGENE 6 transfection reagent at a ratio of 1:3 (DNA:FuGENE 6) and adhering to the manufacturer's instructions. Experiments were performed at 48 h post-transfection, unless otherwise indicated.

### Cell lysis

Cells expressing WT or mutant SR-BI were washed twice in cold PBS on ice and lysed in RIPA buffer containing protease inhibitors for 10 min on ice. Cell lysates were cleared from cellular debris by centrifugation at 6010 *g* for 10 min at 4°C. Protein concentrations of lysates were obtained by the Lowry method (25).

### Immunoblot analysis of SR-BI expression

Cellular lysates (10 μg) were combined with an equal volume of 2× Laemmli buffer [4% SDS, 20% glycerol, 120 mM Tris-HCl (pH 6.8), 0.005% bromophenol blue, and 10% β-mercaptoethanol], separated by 10% SDS-PAGE, wet transferred onto nitrocellulose membranes, and incubated with one of the following antibodies: anti-C-terminal region of SR-BI (1:5,000), anti-GAPDH (1:5,000), or anti-rabbit-IgG-HRP (1:10,000). Bands were visualized on a ChemiDoc system (BioRad), and band intensities were quantified on ImageJ (NIH) software.

### SR-BI cell surface biotinylation

Cells expressing WT or mutant SR-BI were incubated with 1 mg/ml nonmembrane-permeable biotinylation reagent (EZ-Link Sulfo-NHS-LC-Biotin) in PBS for 1 h at 4°C. Biotinylated proteins in a 100 μL aliquot of cell lysate (mean ± SEM of the actual protein amount = 96.1 ± 3.0 μg for 30 total samples) were pulled down on streptavidin beads, eluted in 2× Laemmli buffer containing 10% β-mercaptoethanol, and separated by SDS-PAGE, as previously described (12). SR-BI was detected by immunoblot using the C-terminal targeting SR-BI antibody.

### SR-BI oligomerization analysis

Cells expressing WT or mutant SR-BI were harvested in cold PBS containing protease inhibitors and briefly sonicated for four cycles. Cell lysates (10 μg) were combined with perfluorooctanoic acid (PFOA) sample treatment buffer (5% PFOA, 100 mM Tris base, 20% glycerol, 0.005% bromophenol blue without β-mercaptoethanol) and separated by PFOA-PAGE (26) with minor modifications, as previously described (27). SR-BI was detected by immunoblot using the SR-BI C-terminal-targeting antibody.

### HDL binding, cellular association, and cholesteryl ester uptake

Human HDL was labeled with CHE and iodinated with [^125^I]dilactitol tyramine using established protocols (28). The initial specific activities for the double-radiolabeled HDL preparation in disintegrations per minute/nanogram protein (mean ± SEM) were [^125^I] = 206.9 ± 86.2 and [^3^H] = 246.7 ± 42.4. HDL binding, cellular association, and cholesteryl ester uptake of double-radiolabeled HDL particles were measured at 4, 37, and 37°C, respectively, as previously described (29).

### Free cholesterol efflux

Cells expressing WT or mutant SR-BI were [^3^H]cholesterol labeled and used to measure free cholesterol efflux as previously described (27) with slight modifications. Namely, the equilibration step was performed in DMEM/0.2% BSA, and the HDL incubation was in serum-free DMEM.

### Cholesterol oxidase sensitivity assay

Cells expressing WT or mutant SR-BI were [^3^H]cholesterol labeled, treated with cholesterol oxidase, and subjected to lipid separation, as previously described (27).

### DiI-LDL and DiI-HDL binding and internalization

Cells expressing WT-SR-BI or R174C-SR-BI were incubated with 0, 10, or 25 μg/ml DiI-LDL or DiI-HDL for 1.5 h at 4°C (after prechilling the plate at 4°C for 10 min) or 37°C. Cells were washed in ice-cold PBS, harvested by pipetting in PBS/0.5% BSA, centrifuged at 300 *g*, and resuspended in PBS/0.5% BSA for analysis on an AccuriC6 flow cytometer (BD Biosciences). LDL or HDL internalization was calculated by subtracting the mean fluorescence intensity (MFI) of lipoprotein binding at 4°C from the MFI measured at 37°C.

### Data normalization and statistical analysis

Raw data values were normalized to WT levels of activity (WT-SR-BI = 100%). One-way ANOVA and Dunnett's multiple comparisons tests were used to compare three or more groups. For experiments with two independent variables, two-way ANOVA and Tukey multiple comparisons tests were used to determine statistical significance. Normalized or raw data values are expressed as the mean ± SEM, where ∗*P* < 0.05, ∗∗*P* < 0.01, and ∗∗∗*P* < 0.001 versus WT-SR-BI.

## Results

### R174C-SR-BI is a rare human variant of SR-BI identified in an individual with high HDL-cholesterol

**Fig. 1:**
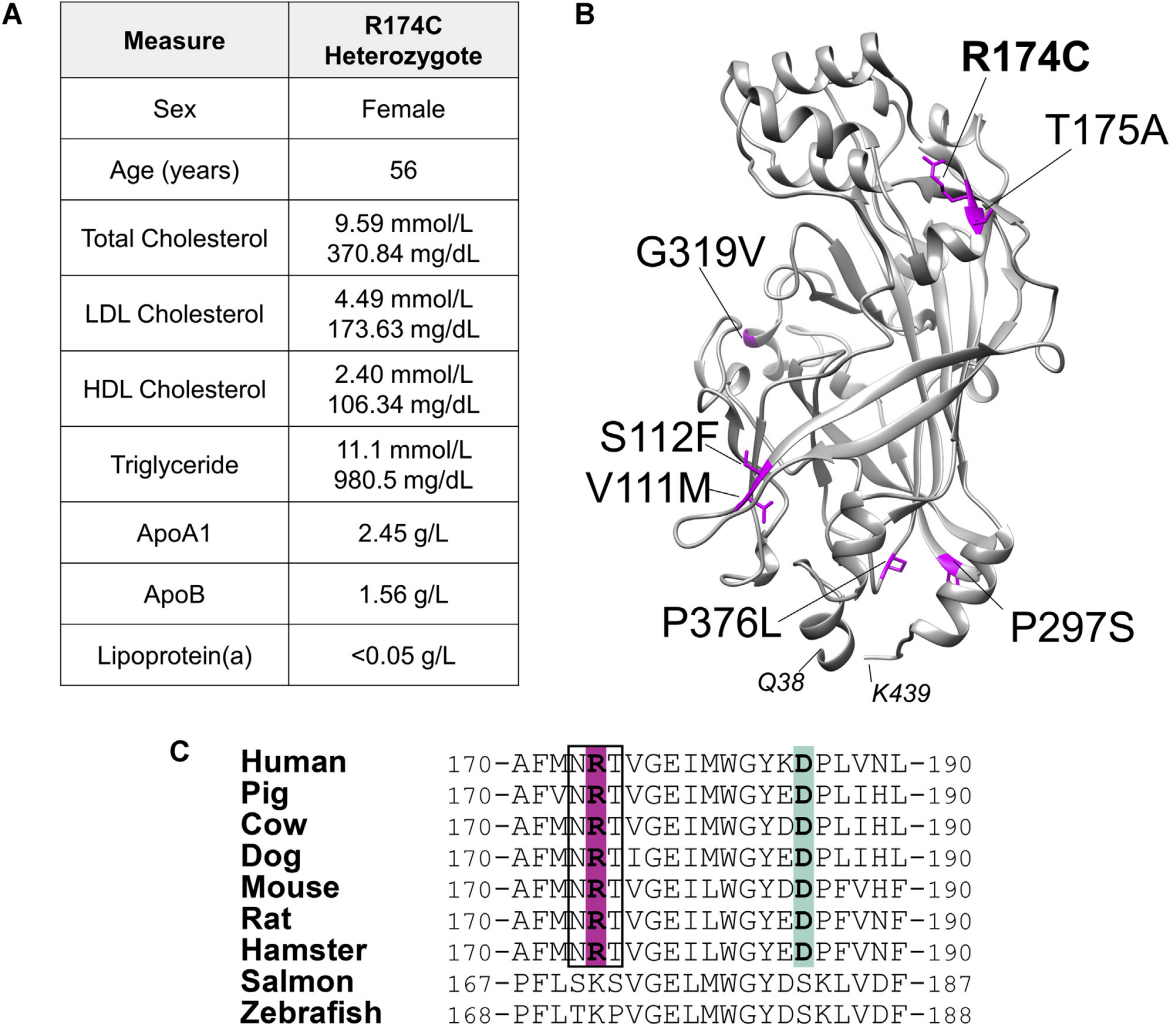
R174C-SR-BI is a rare human variant of SR-BI identified in an individual with high HDL-cholesterol. A: Characteristics and lipid profile of the R174C-SR-BI heterozygous individual. B: A homology model of the extracellular domain of human SR-BI (residues 38–439) was generated using Modeller software, and residues that correspond to sites of known human variants are colored in pink. C: Multiple species sequence alignments of SR-BI were generated by the MUltiple Sequence Comparison by Log-Expectation algorithm. Arg-174 and Asp-185 (possible ion pair) are highlighted in magenta and light green, respectively, and the boxed region represents a conserved N-linked glycosylation sequence (NXT). SR-BI, scavenger receptor class B type I.

The index patient was referred to the lipid genetics clinic at age 56 with a long-standing history of hyperlipidemia. The patient was free of any cardiovascular symptoms and had no history of diabetes, hypertension, or cigarette smoking and took no lipid-lowering medications. However, they drank ∼1 alcoholic beverage per day. The patient's mother died of a stroke at age 65, and the patient's sister had a myocardial infarction at age 40. On physical examination, the patient's blood pressure was 120/70 mm Hg, and no physical signs of hyperlipidemia were observed. Physical examination was unremarkable. Fasting blood lipid profile revealed a total cholesterol of 371 mg/dl (normal, <200 mg/dl) or 9.59 mmol/l (normal, <5.2 mmol/l), triglyceride of 981 mg/dl (normal, <175 mg/dl) or 11.1 mmol/l (normal, <2.0 mmol/l), HDL-cholesterol of 106 mg/dl (normal female range, 45–70 mg/dl) or 2.40 mmol/l (normal female range, 1.1–1.8 mmol/l), LDL-cholesterol of 174 mg/dl (normal range, <130 mg/dl) or 4.49 mmol/l (normal range, <3.5 mmol/l), apoA1 of 2.45 g/l (normal range, 1.0–1.4 g/l), apoB of 1.56 g/l (normal range, 0.8–1.2 g/l), and lipoprotein(a) measured using nephelometry (CSL Behring, Ottawa, Ontario, Canada) was <0.05 g/l (normal range, <0.30 g/l). A carotid ultrasound identified a large 1.5 by 2.0 cm calcified plaque along the posterior wall of the right internal carotid artery. No plaque or intima medial thickening was seen in the left carotid artery. Patient characteristics are summarized in Fig. 1A.

Sequencing of DNA using a targeted next-generation sequencing panel revealed two heterozygous variants: c.520C>T in *SCARB1* (SR-BI p.R174C) and c.193C>T in *LIPC* [gene encoding hepatic lipase (LIPC) p.R65X]. The *SCARB1* variant had an MAF of 0.00004942 in ExAC and a scaled CADD score of 26.5, which strongly predicted pathogenicity. The *LIPC* variant had an MAF of 0.00001647 in ExAC and a scaled CADD score of 33, which also strongly predicted pathogenicity.

A homology model of SR-BI was generated, based on the X-ray crystal structure of the luminal domain of lysosomal integral membrane protein 2 (30), to show the predicted location of R174C in relation to the other identified human SR-BI variants (Fig. 1B). Arg-174 is predicted to reside within a β-strand (31, 32). The amino acid sequences of SR-BI in humans and multiple other species were aligned using the MUltiple Sequence Comparison by Log-Expectation algorithm (33). The amino acid sequence of the region surrounding Arg-174 (residues 170–190) is shown (Fig. 1C). Arg-174 is highly conserved across various species, except in lower vertebrates (salmon and zebrafish), which have a conservative substitution of lysine (K) for Arg-174. This region also contains an N-linked glycosylation motif (NXT, where X = Arg-174) that is likely glycosylated (34). The amino acid that immediately follows, Thr-175, is the site of another human variant (T175A) that was similarly identified in individuals with elevated HDL-cholesterol levels (17).

### R174C-SR-BI is expressed at the cell surface of COS-7 cells

**Fig. 2:**
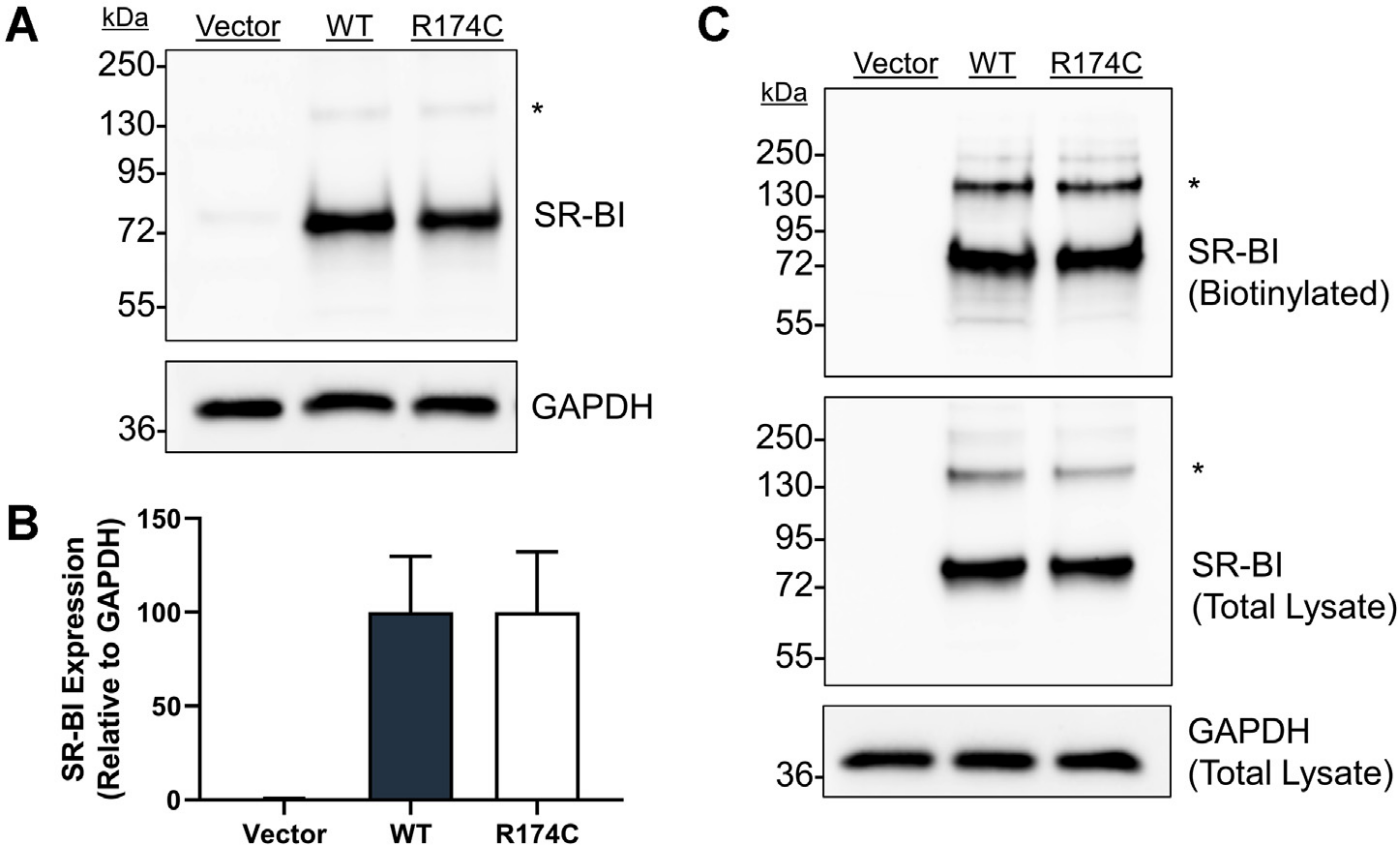
R174C-SR-BI is expressed at the cell surface of COS-7 cells. A: Immunoblot analysis was performed on cell lysates (10 μg) of COS-7 cells transiently transfected with WT-SR-BI or R174C-SR-BI to assess total SR-BI expression. An asterisk (∗) indicates possible SR-BI dimer bands. GAPDH expression was probed as a loading control. B: Densitometry was performed using ImageJ software to quantify SR-BI expression relative to GAPDH. C: Cell surface SR-BI expression (in 100 μl total lysate) was measured following sulfo-NHS-LC biotinylation. Equal volumes of total lysate (10 μl) were loaded to probe for GAPDH, in order to account for small differences in protein loading onto streptavidin beads, and total SR-BI, in order to account for potential differences in overall SR-BI expression. Immunoblots in panels A and C were performed under reducing conditions. Data in panel B are represented as the mean ± SEM of three independent transfections (n = 3), relative to average WT-SR-BI expression levels (WT = 100%). As determined by one-way ANOVA and Dunnett's multiple comparisons tests, no statistical significance was observed between the means for WT-SR-BI and R174C-SR-BI. The immunoblots in panels A and C are each representative of three independent transfections (n = 3). SR-BI, scavenger receptor class B type I.

COS-7 cells serve as an appropriate model system for measuring SR-BI function at the cellular level because of their lack of detectable endogenous SR-BI expression. Cells were transiently transfected with empty plasmid vector (pcDNA3) or human WT-SR-BI or R174C-SR-BI. At 48 h post-transfection, SR-BI expression was analyzed. Immunoblot analysis of cleared cell lysates showed no statistically significant differences in total protein expression of WT-SR-BI and R174C-SR-BI (Fig. 2A), which was quantified by densitometry (Fig. 2B). No change in migration patterns was observed for mutant SR-BI by SDS-PAGE, suggesting that glycosylation status is unaltered in the presence of R174C-SR-BI (supplemental Fig. S1). In order to measure cell surface expression levels of SR-BI, we first biotinylated cell surface proteins using EZ-Link Sulfo-NHS-LC-Biotin, a nonmembrane-permeable biotinylation reagent, before enrichment of biotinylated proteins by pull-down assay using streptavidin-coated beads. Immunoblot analysis of biotinylated proteins indicated that WT-SR-BI and R174C-SR-BI cell surface expression levels are not statistically significantly different (Fig. 2C).

### Cholesterol transport functions of R174C-SR-BI are impaired in COS-7 cells

**Fig. 3:**
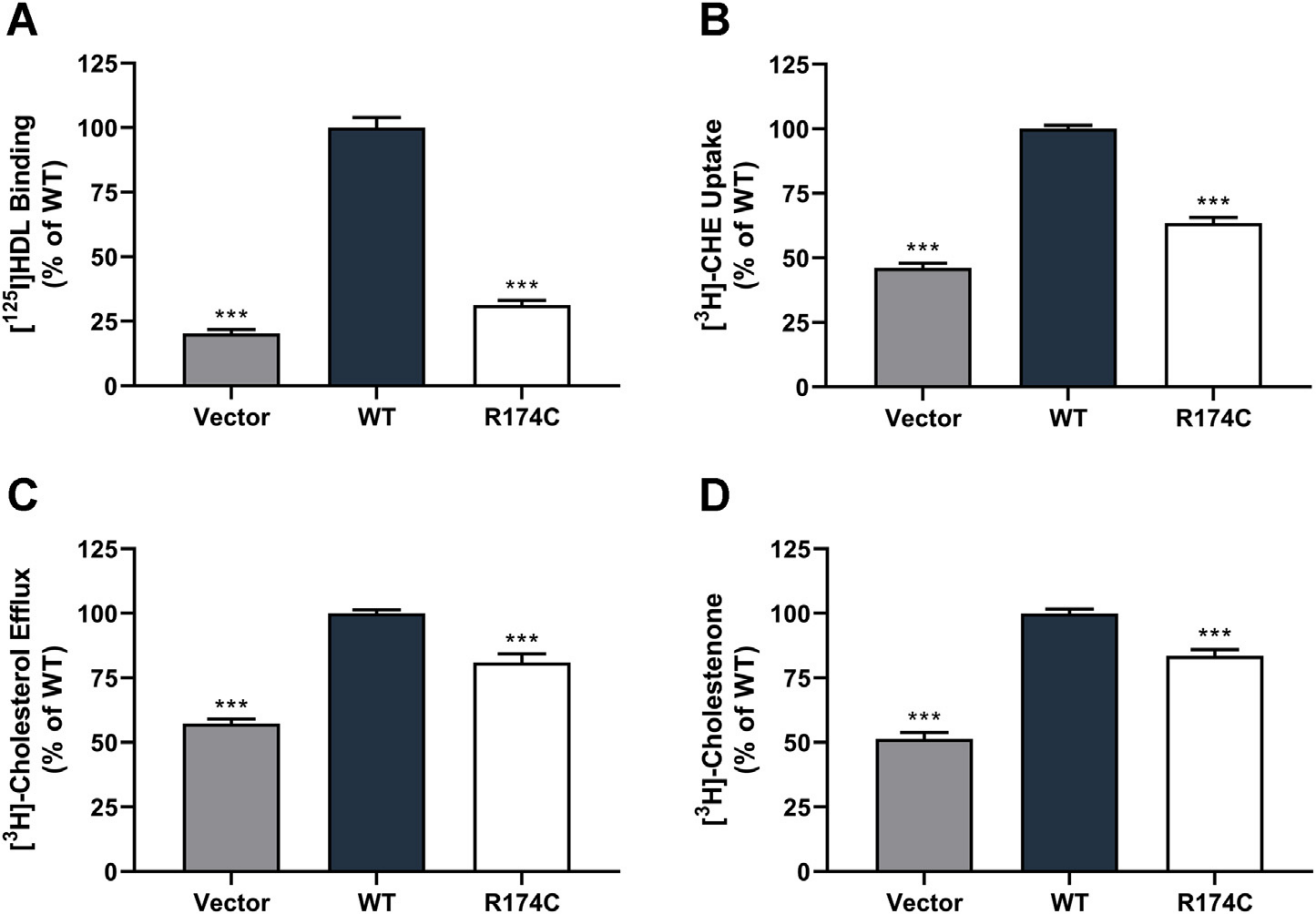
Cholesterol transport functions of R174C-SR-BI are impaired in COS-7 cells. COS-7 cells transiently expressing WT-SR-BI or R174C-SR-BI were incubated with double-radiolabeled human HDL (10 μg/ml) for 1.5 h at 4°C or 37°C to measure (A) [^125^I]-HDL binding and (B) CHE uptake. Raw values (expressed as mean ± SEM) for HDL binding were 4.5 ± 0.4 ng/mg protein (empty vector) and 22.7 ± 1.0 ng/mg protein (WT-SR-BI). For CHE uptake, the raw values were 40.4 ± 5.3 ng/mg protein (empty vector) and 85.6 ± 8.2 ng/mg protein (WT-SR-BI). C: To measure free cholesterol efflux, COS-7 cells were transiently transfected with WT-SR-BI or R174C-SR-BI, prelabeled with [^3^H]cholesterol, and incubated with human HDL (50 μg/ml) for 4 h at 37°C. D: COS-7 cells transiently expressing WT-SR-BI or R174C-SR-BI were prelabeled with [^3^H]cholesterol and incubated with exogenous cholesterol oxidase for 4 h at 37°C. Following lipid extraction, cholesterol species were analyzed by thin-layer chromatography. All data are represented as the mean ± SEM of three to four independent transfections (n = 3–4, with three to four replicates) and are expressed as a percentage of WT-SR-BI activity. As determined by one-way ANOVA, ∗∗∗*P* < 0.001 versus WT-SR-BI. CHE, [^3^H]cholesteryl hexadecyl ether; SR-BI, scavenger receptor class B type I.

To test our hypothesis that cholesterol transport functions are impaired in the presence of the human variant, we measured HDL binding, HDL cell association, and cholesteryl ester uptake in COS-7 cells expressing WT-SR-BI or R174C-SR-BI. The cells were incubated for 1.5 h at 4°C or 37°C with 10 μg/ml HDL labeled with [^125^I] and CHE, after which, they were processed as described above. Results were normalized to WT-SR-BI levels, which produced the same trends as the raw data values (supplemental Table S1). Normalized [^125^I]-HDL binding was decreased to 31.3% of WT-SR-BI levels in the presence of R174C-SR-BI (Fig. 3A). Likewise, uptake of CHE was reduced to 63.5% of WT levels with the human variant (Fig. 3B). Cholesterol efflux from COS-7 cells expressing SR-BI and prelabeled with [^3^H]cholesterol was measured following a 4 h incubation with 50 μg/ml HDL at 37°C. Cholesterol efflux was decreased to 80.9% relative to WT-SR-BI levels (Fig. 3C). We performed an additional functional assay for SR-BI that is independent of HDL. Since SR-BI is able to redistribute pools of free cholesterol within the plasma membrane, we performed a cholesterol oxidase sensitivity assay to determine the accessibility of membrane cholesterol. COS-7 cells expressing SR-BI and prelabeled with [^3^H]cholesterol were treated with 0.5 U/ml cholesterol oxidase for 4 h at 37°C. In the presence of R174C-SR-BI, membrane cholesterol was less accessible to the extracellular oxidase, as [^3^H]cholestenone production was reduced to 83.5% of WT-SR-BI levels (Fig. 3D).

### DiI-LDL internalization is reduced in the presence of R174C-SR-BI

**Fig. 4:**
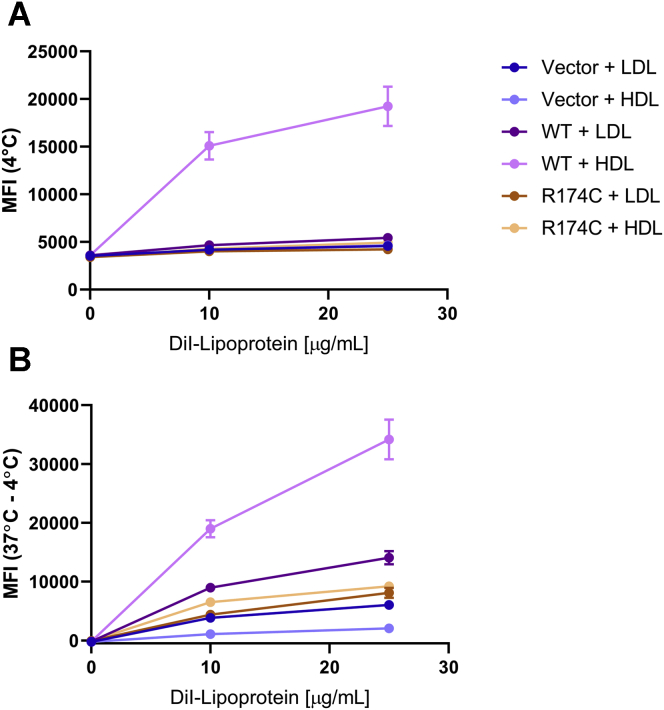
DiI-LDL internalization is reduced in the presence of R174C-SR-BI. COS-7 cells transiently transfected with empty vector, WT-SR-BI, or R174C-SR-BI were incubated with the indicated concentrations of DiI-LDL or DiI-HDL for 1.5 h at 4°C or 37°C. A: Mean fluorescence intensities (MFI) of DiI-LDL and DiI-HDL binding at 4°C are shown. B: DiI-LDL and DiI-HDL internalization were calculated as the differences between MFI at 37°C and MFI at 4°C that can be attributed to lipoprotein binding. Statistically significant differences, as determined by two-way ANOVA, are presented in Supplemental Table S2. Data represent the mean ± SEM of three independent transfections (n = 3) performed in duplicate.

In light of recent reports that SR-BI facilitates LDL transcytosis across the endothelium (35, 36), we assessed the ability of COS-7 cells expressing WT-SR-BI or R174C-SR-BI to facilitate binding and internalization of fluorescently labeled DiI-LDL. The cells were treated with 0, 10, or 25 μg/ml DiI-LDL (or DiI-HDL) for 1.5 h at 4°C or 37°C, and the MFIs of DiI-labeled lipoprotein binding (at 4°C) and internalization (37 − 4°C) were measured by flow cytometry. Raw MFI values and statistically significant differences are shown in supplemental Table S2. DiI-LDL binding was not statistically significantly increased upon overexpression of WT-SR-BI or R174C-SR-BI (Fig. 4A). We observed an increase in DiI-LDL internalization in the presence of WT-SR-BI, but DiI-LDL internalization was statistically significantly decreased with R174C-SR-BI (Fig. 4B). Corroborating our previous findings (Fig. 3A, B), DiI-HDL binding and internalization were statistically significantly decreased in the presence of R174C-SR-BI, as compared with WT-SR-BI (Fig. 4A, B).

### Arg-174 and aspartate-185 may interact through a salt bridge

**Fig. 5:**
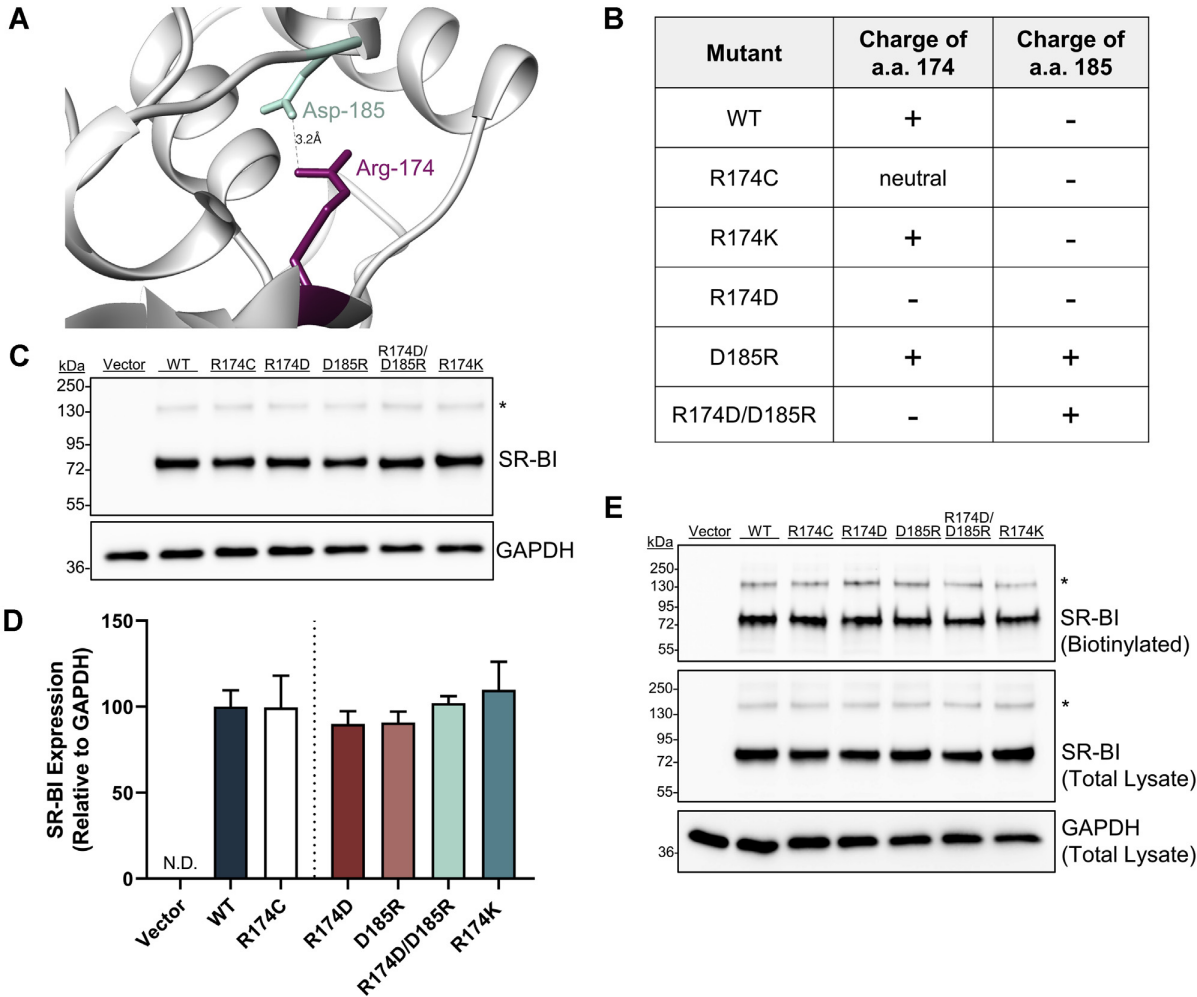
Arginine-174 and aspartate-185 may interact through a salt bridge. A: Homology modeling suggests a possible salt bridge interaction between residues Arg-174 (magenta) and Asp-185 (light green) with an approximate distance of 3.2 Å, as calculated by Chimera software. B: Additional mutant constructs were generated to disrupt or retain the putative salt bridge. C: SR-BI immunoblot analysis was performed on cell lysates (10 μg) from COS-7 cells transiently transfected with empty vector or mutant SR-BI. D: SR-BI expression was quantified relative to GAPDH loading control by densitometry. E: Cell surface levels of SR-BI (in 100 μl total lysate), following sulfo-NHS-LC biotinylation of transiently transfected COS-7 cells, were determined by immunoblot analysis. The additional mutants are colored as follows: salt bridge-disrupting mutants (R174D-SR-BI and D185R-SR-BI) in dark red and light red, the charge swap mutant (R174D-SR-BI/D185R-SR-BI) in light teal, and the conservative mutant (R174K-SR-BI) in dark teal. Immunoblots in panels C and E were performed under reducing conditions. Data in panel D represent the mean ± SEM of three independent transfections (n = 3), relative to average WT-SR-BI expression levels (WT = 100%). As determined by one-way ANOVA, no statistical significance was observed between the means for WT-SR-BI and mutant SR-BI receptors. The immunoblots in panels C and E are each representative of three independent transfections (n = 3). SR-BI, scavenger receptor class B type I.

A closer examination of our homology model suggests the possibility of a salt bridge that may link Arg-174 and aspartate-185 (Asp-185) with an approximate distance of 3.2 Å between side chains (Fig. 5A). This distance is within the ≤4 Å limit for ion pairs, as defined by Barlow and Thornton (37). We predicted that loss of a stabilizing salt bridge could be one mechanism by which R174C-SR-BI is dysfunctional. To test if the putative salt bridge is required for SR-BI function, we generated an additional set of mutations that would disrupt potential salt bridge interactions between Arg-174 and Asp-185 (Fig. 5B). Specifically, we generated two single-point mutants, R174D-SR-BI and D185R-SR-BI, where the side chains at both positions (174 and 185) would be negative or positive, respectively. As controls, we generated a conservative mutation (R174K), as well as a charge swap double mutant (R174D-SR-BI/D185R-SR-BI) that we predict will maintain normal SR-BI function as the putative salt bridge should remain intact. Expression analysis of the new mutants by immunoblotting (Fig. 5C, D) and cell surface protein biotinylation assays (Fig. 5E) verified that there were no statistically significant changes in the levels of expression of WT and mutant SR-BI constructs.

### Cholesterol transport functions of salt bridge mutants vary by mutation

**Fig. 6:**
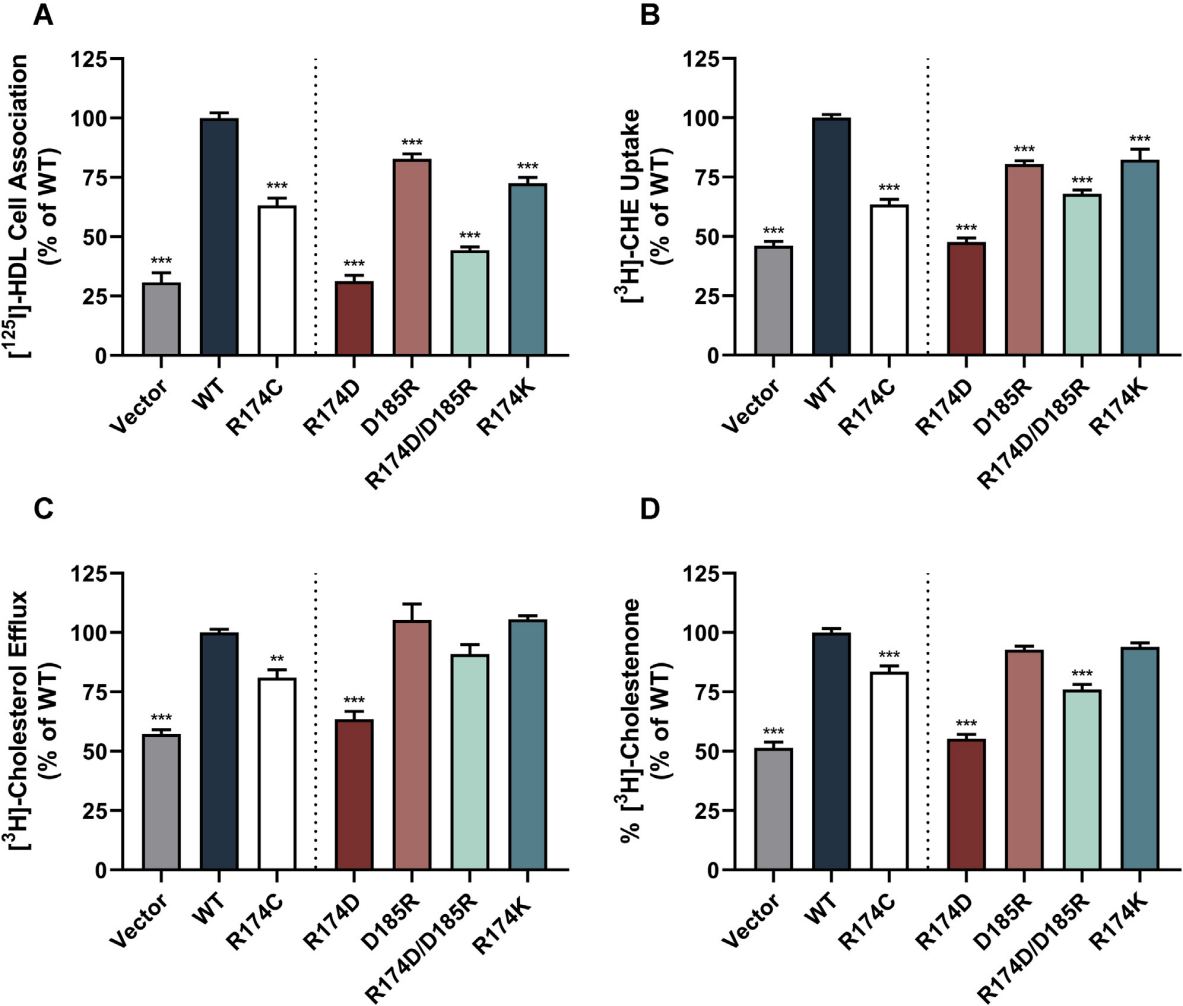
Cholesterol transport functions of salt bridge mutants vary by mutation. COS-7 cells transiently expressing WT or mutant SR-BI were assessed for (A) [^125^I]-HDL cell association, (B) [^3^H]CHE uptake, (C) [^3^H]cholesterol efflux, and (D) [^3^H]cholesterol accessibility. A dotted vertical line separates results for the human variant (previously shown in Fig. 3) from that of additional mutants generated to assess the importance of the putative salt bridge. The additional mutants are colored as follows: salt bridge-disrupting mutants (R174D-SR-BI and D185R-SR-BI) in dark red and light red, the charge swap mutant (R174D-SR-BI/D185R-SR-BI) in light teal, and the conservative mutant (R174K-SR-BI) in dark teal. All data are represented as the mean ± SEM of three independent transfections (n = 3) and are expressed as a percentage of WT-SR-BI activity. As determined by one-way ANOVA, ∗∗∗*P* < 0.001, ∗∗*P* < 0.01, and ∗*P* < 0.05 versus WT-SR-BI. CHE, [^3^H]cholesteryl hexadecyl ether; SR-BI, scavenger receptor class B type I.

In order to test the importance of the putative salt bridge, functional assays were performed as described above. The same trends were observed in raw data values and data normalized to WT-SR-BI levels (supplemental Table S1). Although the conservative R174K-SR-BI mutant led to a decrease in HDL cell association (Fig. 6A) that was comparable to R174C-SR-BI, it led to a less severe reduction in cholesteryl ester uptake (Fig. 6B). The ability of R174K-SR-BI to mediate free cholesterol efflux was unaffected (Fig. 6C); whereas a moderate, but statistically significant, reduction in membrane cholesterol accessibility was observed (Fig. 6D). Mutating Arg-174 to a negatively charged amino acid (R174D-SR-BI) dramatically reduced all SR-BI functions that were tested. Interestingly, mutating Asp-185 to a positive charge (D185R-SR-BI) resulted in only moderate decreases in HDL cell association, cholesteryl ester uptake, and membrane cholesterol accessibility, while having no significant impact on free cholesterol efflux. The effects were strikingly similar to those observed with the conservative mutant, R174K-SR-BI. The charge swap mutant (R174D-SR-BI/D185R-SR-BI) displayed a phenotype that was intermediate to the R174D-SR-BI or D185R-SR-BI mutants alone.

### R174C-SR-BI and salt bridge mutants form dimers and higher order oligomers

**Fig. 7:**
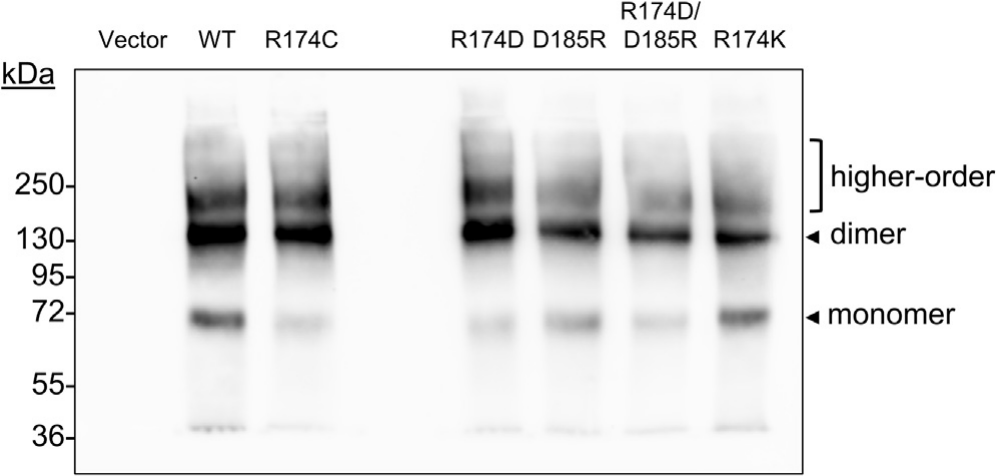
R174C-SR-BI and “salt bridge” mutants form dimers and higher order oligomers. COS-7 cells transiently expressing WT-SR-BI or mutant SR-BI were sonicated, and proteins (10 μg) were separated in the presence of 5% perfluorooctanoic acid under nonreducing conditions for subsequent immunoblot analysis to assess the oligomerization profiles of SR-BI. A representative immunoblot is shown for three independent transfections (n = 3). SR-BI, scavenger receptor class B type I.

SR-BI is reported to form dimers and higher order oligomers, which may play a critical role in its cholesterol transport functions (38, 39, 40, 41, 42). Given that the human variant R174C-SR-BI has reduced ability to mediate cholesterol transport functions, we further tested its ability to form dimers and higher order oligomers using PFOA-PAGE. PFOA is a nondissociative detergent that has been previously used to analyze the oligomeric states of membrane proteins (26), including SR-BI (18). Upon PFOA-PAGE analysis, we observed that R174C-SR-BI and all additional mutants maintained the ability to form dimers and higher order oligomers (Fig. 7). However, there were some differences in the relative abundance of SR-BI monomers, dimers, and higher order oligomers (Fig. 7).

### Electrostatic surface charges are disrupted by R174C-SR-BI

**Fig. 8:**
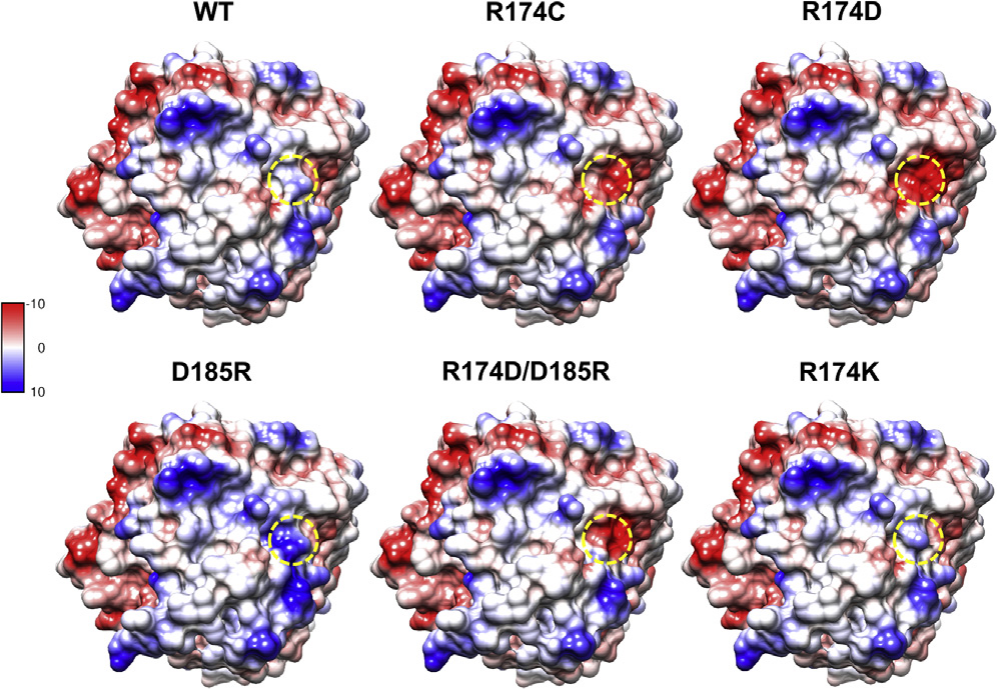
Electrostatic surfaces charges are disrupted by R174C-SR-BI. Arg-174 in WT-SR-BI (top, left) was mutated to cysteine (top, middle) and each of the additional mutants using Chimera software. A top down view of the SR-BI homology model is shown with surface electrostatic coloring, ranging from more negative [red, −10 kcal/(mol ∗ e)] to more positive [blue, 10 kcal/(mol ∗ e)]. The region where Arg-174 occupies and the corresponding regions for R174C-SR-BI and the additional mutants are highlighted in yellow circles. SR-BI, scavenger receptor class B type I.

Mutation of positively charged Arg-174 to a neutral cysteine residue could have an impact on the surface electrostatic charge distribution. SR-BI has been proposed to bind its ligands through a helical bundle and/or electrostatic interactions in the cationic apex region of the receptor (30). The presence of Arg-174 in the apex region (on β-strand 7) may contribute to the overall cationic surface charge of SR-BI, and thus, may be important for HDL binding. The potential effects on surface electrostatic charge distribution induced by the human variant and the additional mutant receptors were assessed. All mutations were generated in the SR-BI homology model using Chimera software (UCSF), and the electrostatic charges were displayed on the surface of the molecule using the Coulombic Surface Charges tool (Fig. 8). Compared with the WT receptor, R174C-SR-BI has less overall cationic surface charges, because of the loss of Arg-174 as well as exposure of negatively charged regions beneath Arg-174. For the remaining mutants, this region appeared to be more negative for R174D-SR-BI and R174D-SR-BI/D185R-SR-BI and more positive or similar to WT with D185R-SR-BI and R174K-SR-BI, respectively. The loss of cationic surface charges appears to be consistent with the degree of loss of function.

## Discussion

Studies have shown that the association between HDL-cholesterol levels and all-cause mortality follows a U-shaped curve, in which both the lowest and highest HDL-cholesterol concentrations are associated with higher mortality (43, 44, 45, 46). In the CANHEART study of individuals in Ontario, Canada, high HDL-cholesterol levels (>70 mg/dl for men and >90 mg/dl for women) were associated with increased hazard of noncardiovascular mortality (44). The R174C-SR-BI heterozygous patient described in this study presented with HDL-cholesterol levels of 106 mg/dl, which is comparable to subjects with the highest HDL-cholesterol levels in the CANHEART study (44). Since the presence of a normal SR-BI allele may compensate for the dysfunctional R174C variant in the heterozygous condition, we might anticipate a further increase in HDL-cholesterol levels for an R174C-SR-BI homozygote. The HDL-cholesterol levels of the R174C-SR-BI heterozygote are comparable to P376L-SR-BI heterozygotes, whose levels are between those of normal SR-BI controls and the P376L-SR-BI homozygote (19). Unfortunately, neither R174C-SR-BI homozygotes were identified in the prior study nor do we have access to any family history to analyze inheritance patterns of this variant. The patient does not have mutations in cholesteryl ester transfer protein or endothelial lipase, two proteins for which genetic deficiency is known to increase HDL-cholesterol levels (47, 48). However, the patient is heterozygous for a mutation in hepatic lipase, namely *LIPC* p.R65X, which predicted early truncation of a nonfunctional protein lacking the active site catalytic triad (49). Total hepatic lipase deficiency in humans can increase HDL-cholesterol (50), while heterozygous carriers of dysfunctional *LIPC* variants do not have a consistent dyslipidemia phenotype (51). Therefore, even though the R174C-SR-BI variant itself has impaired function, the combination of only a single functional copy of *LIPC* and the R174C-SR-BI variant may be necessary to observe the elevation in high HDL-cholesterol levels.

Our studies confirm that, at least in vitro, R174C-SR-BI exhibits partial loss of cholesterol transport functions, including HDL binding, cholesteryl ester uptake, DiI-LDL binding and internalization, free cholesterol efflux, and membrane cholesterol redistribution. Cell surface expression levels and oligomerization patterns were essentially unchanged, indicating they are unlikely mechanisms for R174C-SR-BI dysfunction. We cannot exclude possible contributions from other cholesterol transporters, such as ABCA1 and ABCG1. However, because of negligible expression levels of these cholesterol transporters in COS-7 cells (52, 53), we attribute these functional effects primarily to SR-BI. We have considered several potential structural bases to explain the loss of function of R174C-SR-BI, including glycosylation status, non-native disulfide bond formation, loss of a stabilizing salt bridge, and disruption to the surface charges of SR-BI.

Human SR-BI has nine potential N-linked glycosylation sites in its extracellular domain, whereas murine SR-BI has 11 sites, which all appeared to be N-glycosylated in COS-M6 cells (34). The conserved glycosylation site at position 173 consists of an Asn-X-Thr (NXT) motif. Mutation of Asn-173 to Gln (N173Q) or mutation of Thr-175 to Ala (T175A, another SR-BI human variant) prevented glycosylation, as observed by a slight downward shift (decreased apparent mass) on SDS-PAGE as compared with WT-SR-BI (18, 34). Both N173Q-SR-BI and T175A-SR-BI had reduced cell surface expression and lipid uptake abilities (18, 34). Arg-174 falls in the center position of the NXT motif, where X can be any amino acid except proline. We observed no downward shift on SDS-PAGE for R174C or any of the mutations, suggesting that Asn-173 remains glycosylated. Direct comparison of R174C-SR-BI with T175A-SR-BI on SDS-PAGE confirmed that there was no shift in apparent mass with the human variant (supplemental Fig. S1A). This is perhaps not surprising because oligosaccharyltransferase, the enzyme catalyzing N-linked glycosylation, appears to show no preference for different types of amino acids in the central position of the NXT motif (54). In terms of SR-BI function, the two human mutations (R174C and T175A) lead to distinct effects. While T175A-SR-BI functions are totally impaired, R174C-SR-BI exhibits more of a moderate impact on SR-BI cholesterol transport functions.

Introducing a non-native cysteine at amino acid 174 could result in non-native disulfide bond formation, either within a single SR-BI molecule, between multiple SR-BI molecules, or between SR-BI and other proteins. To address the possibility of non-native intramolecular or intermolecular disulfide formation, we performed immunoblot analysis following SDS-PAGE in the absence of β-mercaptoethanol. Interestingly, we observed less of the monomeric form of R174C-SR-BI than WT-SR-BI, despite no observable increase in higher molecular weight bands (supplemental Fig. S1B) and total SR-BI expression appearing similar in the presence of reducing agent (Fig. 2; supplemental Fig. S1B). This could be an artifact of sample preparation for SDS-PAGE, resulting in disulfide bond scrambling and potential antibody epitope masking. Indeed, WT-SR-BI may contain at least two free cysteines within the extracellular domain (55), which could bind to the non-native cysteine residue during sample preparation. In an attempt to avoid epitope masking, we probed with antibodies targeting three different regions on SR-BI [the C-terminal region (within residues 450–509), the near-C-terminal extracellular domain (residues 230–380), and the near-N-terminal extracellular domain (residues 50–150)], all of which showed a decrease in monomeric R174C-SR-BI (data not shown). Further, incubation or cell lysis in the presence of free cysteine blocking molecules (iodoacetamide and N-biotinylaminoethyl methanethiosulfonate-biotin) did not prevent the decrease in monomer observed with R174C-SR-BI under nonreducing conditions (data not shown). Surprisingly, other mutants also showed an apparent decrease in the relative expression of monomeric SR-BI (particularly R174D-SR-BI; Fig. 7), but this result cannot be explained by disulfide bond scrambling, as the other mutants likely have the same number of free cysteine residues as WT-SR-BI. Future studies will be needed to confirm if antibody recognition is disrupted by the mutants, or alternatively, if the monomeric form of the mutant receptors becomes more readily degraded.

Based on homology modeling, it appears that the guanidinium group of Arg-174 and carboxylate group of Asp-185 on SR-BI could potentially interact to form a salt bridge. However, our results suggest that a disrupted salt bridge may not be responsible for the cholesterol transport defects observed with R174C-SR-BI. The dramatic decrease in function with R174D-SR-BI (one of the salt bridge-disrupting mutants) is consistent with a functional role for the salt bridge. However, the D185R-SR-BI mutant did not demonstrate a similar decrease in function, suggesting that the putative salt bridge is dispensable for function. Further, mutational analyses of the SR-BI homology model suggest that the charge-swapped residues remain within close enough proximity to facilitate a salt bridge (<4 Å), yet this mutant demonstrates impaired function. If, however, a salt bridge does exist, it is possible that it does not contribute much to structural stability, as ion pairs that are fully solvent accessible may contribute less to structural stability than those that are buried (56). Availability of a high-resolution structure for the extracellular domain of SR-BI will help resolve these issues related to the importance of a salt bridge or other neighboring structural features that may change upon mutation of Arg-174.

An alternative possibility is that Arg-174 may be primarily solvent exposed. In this case, we speculated that simply the positive charge of Arg-174 could be important for its function, particularly because of the dramatic impact of the arginine-to-aspartate mutation on SR-BI function. Upon applying Coulombic surface coloring to the surfaces of the SR-BI homology models for WT-SR-BI and R174C-SR-BI, as well as the additional mutants, we noticed clear differences in the surface charge distributions. Interestingly, the overall surface charges in this region (Fig. 8) seem to mirror the effects on function (Fig. 6), with the mutants having more negatively charged surfaces generally exhibiting greater decreases in function. One exception for this is D185R-SR-BI, which seems to have greater positive charge on the surface of the receptor. However, difficulties in accommodating such a dramatic mutation and minimizing steric clashing could explain the decreased function of D185R-SR-BI. Notably, Arg-174 was found to be present within a cholesterol binding motif (cholesterol consensus motif) in SR-BI (57). Within cholesterol binding motifs, Arg may interact with the hydroxy group of cholesterol via hydrogen bonding and/or ion-dipole interactions, as previously suggested (58, 59, 60). Thus, Arg-174 could potentially facilitate direct interactions with cholesterol on HDL particles. Two additional SR-BI human variants are listed in the gnomAD database: R174H-SR-BI and R174S-SR-BI (61). Their functions have not been reported; however, they are predicted to be benign/tolerated and possibly damaging/deleterious by PolyPhen/Sorting Intolerant From Tolerant, respectively (61).

To date, relatively few studies have identified and characterized the functions of human SR-BI genetic variants. In the current study, we have characterized a rare human variant of SR-BI (R174C) that was previously identified in a patient with high HDL-cholesterol levels, and this variant had reduced ability to perform cholesterol transport functions in cells. The underlying mechanism of its reduced function does not appear to be through loss of glycosylation, non-native disulfide bond formation, impaired oligomerization, or loss of a stabilizing salt bridge. It may, however, involve disruptions to the surface electrostatic charges of SR-BI, resulting in a decrease in the net positive charge, which could impact its ability to bind to HDL particles. Although SR-BI primarily binds and internalizes HDL (Fig. 4), there was even less LDL internalized in the presence of the human variant. In this context, R174C-SR-BI may play a protective role against atherosclerosis by inhibiting LDL transcytosis across the endothelium. Future investigations will seek to understand the role of R174C-SR-BI across different tissue types and in whole-body cholesterol clearance. Studies such as this improve our understanding of the structural features of SR-BI that drive its ability to function in cholesterol transport and highlight the importance of looking beyond simple HDL-cholesterol measurements to determine cardiovascular disease risk.

### Data availability

All data reported in this study are located within the main text or supplemental data and are available upon request to Dr Daisy Sahoo (Medical College of Wisconsin, dsahoo@mcw.edu).

## Supplemental data

This article contains supplemental data.

## Conflict of interest

The authors declare that they have no conflicts of interest with the contents of this article.
