# Supplementary material for: Human variant of scavenger receptor BI (R174C) exhibits impaired cholesterol transport functions
Source: J Lipid Res. 2021 Feb 9;62:100045. doi: 10.1016/j.jlr.2021.100045 (PMC7985710; doi:10.1016/j.jlr.2021.100045)
Supplement: Supplemental Figure S1 and Supplemental Tables S1 & S — 2 [file mmc1.docx]

**SUPPLEMENTAL TABLE S1**


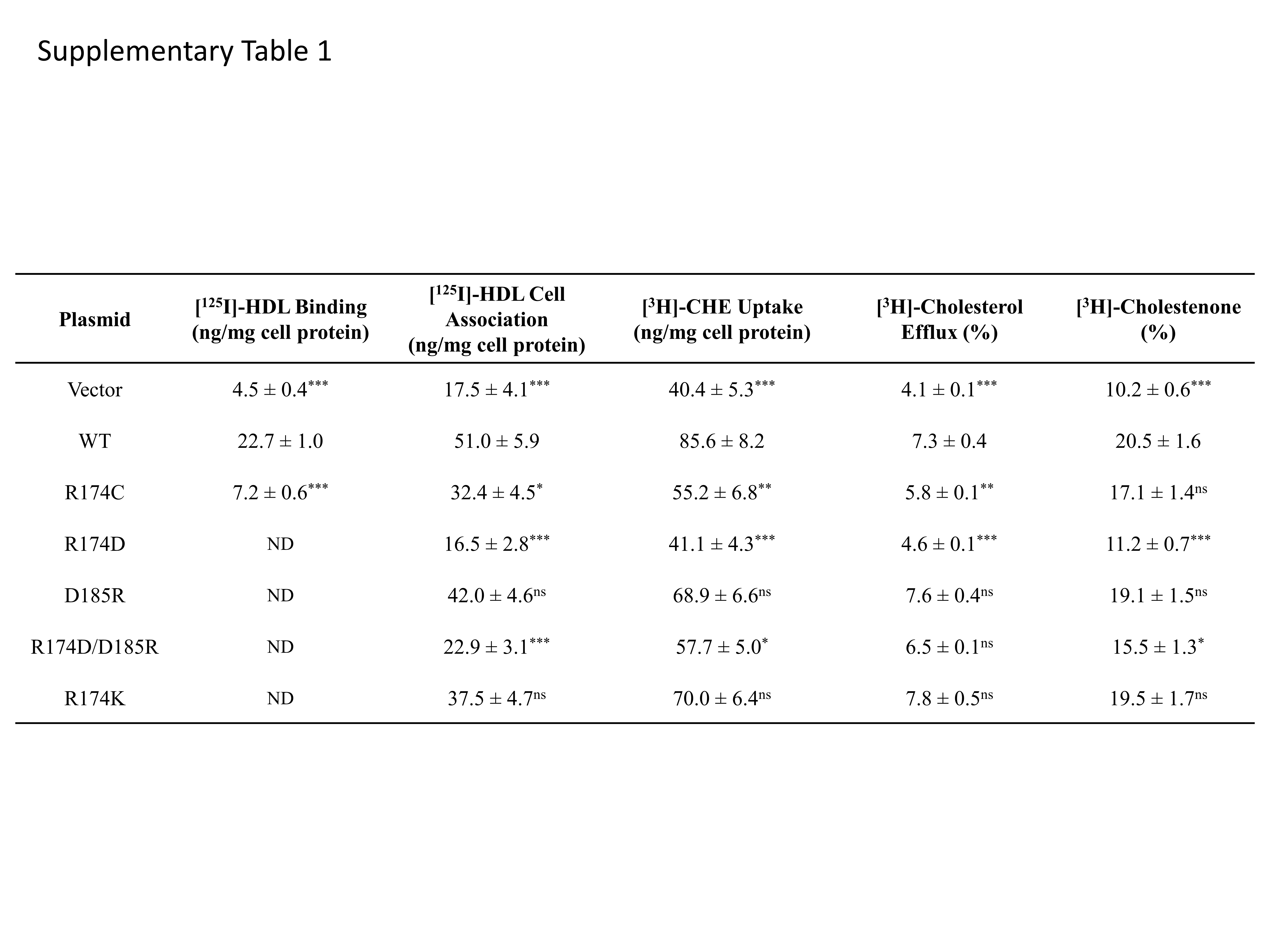


**Supplemental Table S1: Raw data from SR-BI functional assays.** Raw data (units in parentheses) are represented as the mean ± SEM of three to four independent transfections (n = 3-4). ND indicates that [^125^I]-HDL binding was not determined for the specified mutant. By one-way ANOVA and Dunnett’s multiple comparisons tests, ***P<0.001, **P<0.01, *P<0.05, and ns = no significance (P>0.05) versus WT-SR-BI levels.

**SUPPLEMENTAL TABLE S2**


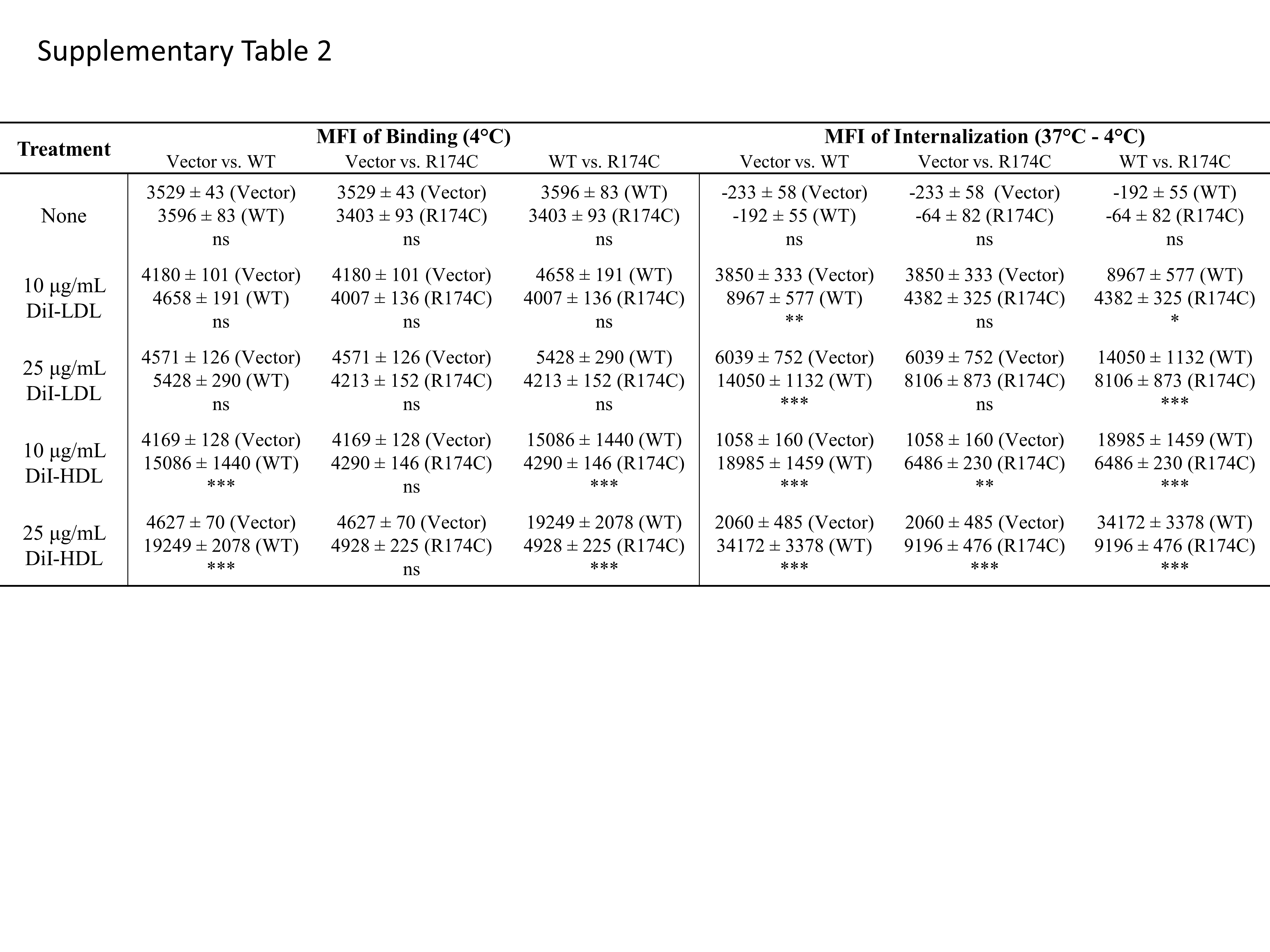


**Supplemental Table S2: Raw data from DiI-LDL and DiI-HDL binding and internalization assays.** Raw data (mean fluorescence intensities) are represented as the mean ± SEM of three independent transfections (n = 3). By two-way ANOVA and Tukey’s multiple comparisons tests, ***P<0.001, **P<0.01, *P<0.05, and ns = no significance (P>0.05) versus WT-SR-BI levels.

**SUPPLEMENTAL FIGURE S1**


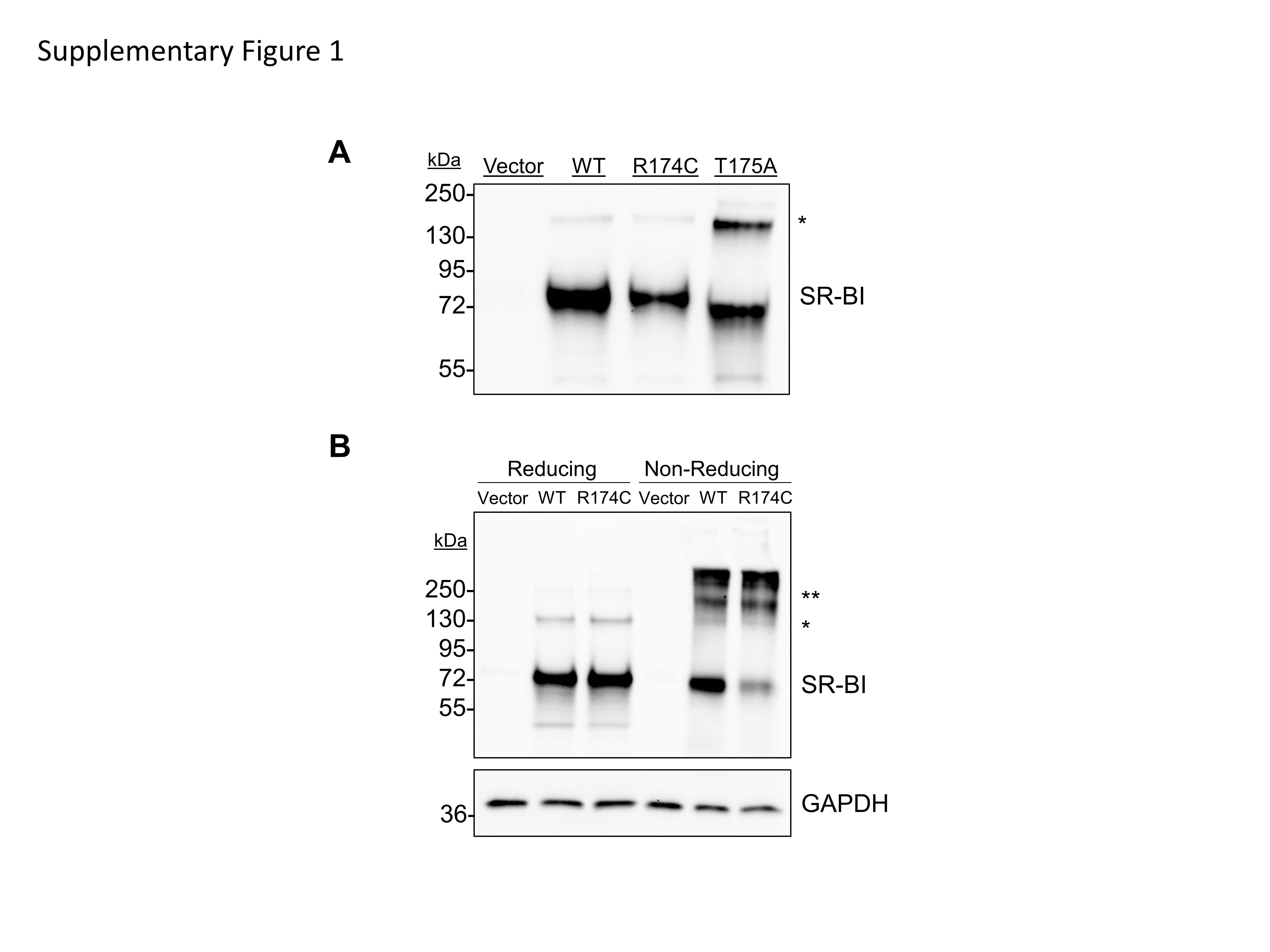


**Supplemental Figure S1: R174C-SR-BI is fully glycosylated, and fewer R174C-SR-BI monomers are detectable under non-reducing conditions.** (A) In order to detect possible shifts in apparent molecular weight upon loss of glycosylation, cell lysates (10 μg) of COS-7 cells expressing empty vector, WT-, R174C-, or T175A-SR-BI were separated by 10% SDS-PAGE under reducing conditions. Membranes were probed with anti-C-terminal SR-BI antibody. (B) Cell lysates (10 μg) of COS-7 cells transiently transfected with empty vector, WT-, or R174C-SR-BI were combined with 2X Laemmli buffer with (reducing) or without (non-reducing) 10% β-mercaptoethanol and separated by 10% SDS-PAGE. Membranes were probed with anti-C-terminal SR-BI or anti-GAPDH antibodies. Immunoblots are representative of three independent transfections (n = 3), and identical results were achieved with two additional antibodies targeting SR-BI’s near-N-terminal extracellular region (ab52629; Abcam) and near-C-terminal extracellular region (NB400-134; Novus Biologicals). Asterisks indicate (*) possible SR-BI dimer and (**) higher-order oligomers/SR-BI-containing multiprotein complexes/aggregates.
